# *RARS1*‐related hypomyelinating leukodystrophy‐9 (HLD‐9) in two distinct Iranian families: Case report and literature review

**DOI:** 10.1002/mgg3.2435

**Published:** 2024-04-15

**Authors:** Sajjad Biglari, Hassan Vahidnezhad, Mohammad Amin Tabatabaiefar, Hamid Reza Khorram Khorshid, Emran Esmaeilzadeh

**Affiliations:** ^1^ Department of Genetics and Molecular Biology, School of Medicine Isfahan University of Medical Sciences Isfahan Iran; ^2^ Division of Human Genetics Children's Hospital of Philadelphia Philadelphia Pennsylvania USA; ^3^ Center for Applied Genomics Children's Hospital of Philadelphia Philadelphia Pennsylvania USA; ^4^ Department of Pediatrics University of Pennsylvania, Perelman School of Medicine Philadelphia Pennsylvania USA; ^5^ Genetics Research Center University of Social Welfare and Rehabilitation Science Tehran Iran; ^6^ Fetal Health Research Center Hope Generation Foundation Tehran Iran

**Keywords:** exome sequencing, leukodystrophy and hypomyelination, *RARS1*

## Abstract

**Background:**

Hypomyelinating leukodystrophy‐9 (HLD‐9) is caused by biallelic pathogenic variants in *RARS1*, which codes for the cytoplasmic tRNA synthetase for arginine (ArgRS). This study aims to evaluate the clinical, neuroradiological, and genetic characteristics of patients with RARS1‐related disease and determine probable genotype–phenotype relationships.

**Methods:**

We identified three patients with *RARS1* homozygous pathogenic variants. Furthermore, we performed a comprehensive review of the literature.

**Results:**

Homozygous variants of *RARS1* (c.2T>C (p.Met1Thr)) were identified in three patients with HLD‐9. Clinical symptoms were severe in all patients. Following the literature review, thirty HLD‐9 cases from eight studies were found. The 33 patients' main symptoms were hypomyelination, language delay, and intellectual disability or developmental delay. The mean age of onset for HLD9 in the group of 33 patients with a known age of onset was 5.8 months (SD = 8.1). The interquartile range of age of onset was 0–10 months. Of the 25 variants identified, c.5A>G (p.Asp2Gly) was identified in 11 patients.

**Conclusion:**

Pathogenic variants in *RARS1* decrease ArgRS activity and cause a wide range of symptoms, from severe, early onset epileptic encephalopathy with brain atrophy to a mild condition with relatively maintained myelination. These symptoms include the classic hypomyelination presentation with nystagmus and spasticity. Furthermore, the pathogenicity of the variation c.2T>C (p.Met1Thr) has been shown.

## Graphical abstract

Hypomyelinating leukodystrophy‐9 (HLD‐9) is caused by biallelic pathogenic variants in RARS1. Variants in RARS1 decrease ArgRS activity and cause a wide range of symptoms, from severe, early onset epileptic encephalopathy with brain atrophy to a mild condition with relatively maintained myelination. We reviewed the articles and identified the first three patients with RARS1 homozygous pathogenic variants in Iran.

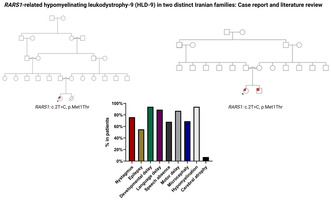

## 1 INTRODUCTION

Leukodystrophies are a broad group of inherited white matter (WM) conditions that can be categorized from many different aspects. Leukodystrophies can be divided into five types based on their pathological alterations and pathogenic mechanisms: myelin disorders, astrocytopathies, leuko‐axonopathies, microgliopathies, and leuko‐vasculopathies (van der Knaap & Bugiani, 2017; van der Knaap et al., 2019). According to this categorization, hypomyelinating leukodystrophies (HLDs) are myelin disorders with impaired myelin production. Although it might not accurately reflect the underlying disease pathomechanism, a classification based on brain magnetic resonance imaging (MRI) pattern recognition is crucial for the diagnostic procedure. Leukodystrophies are hypomyelinating or demyelinating based on MRI (Lynch et al., 2019; Schiffmann & van der Knaap, 2009).

The pathogenesis of various leukodystrophies has been linked to pathogenic variants in genes that encode aminoacyl tRNA (transfer ribonucleic acid) synthetase (aaRS) enzymes in the cytoplasm and/or mitochondria (Diodato et al., 2014; Konovalova & Tyynismaa, 2013). *RARS*1 encodes cytoplasmic arginyl‐tRNA synthetase belonging to class I of the aaRSs (Nafisinia et al., 2017). The aaRSs serve an important role in protein synthesis by ligating each amino acid to its cognate tRNA, which results in the synthesis of proteins in mitochondria and ribosomes (Li et al., 2021).

Since the first HLD, Pelizaeus‐Merzbacher disease (PMD), and its pathology were described in 1885 and 1910, respectively, several diseases characterized by hypomyelination have been discovered through MRI pattern recognition analysis, genetic linkage, and more recently next‐generation sequencing (NGS) techniques (Kevelam et al., 2016; Merzbacher, 1910; Pelizaeus, 1885; van der Knaap et al., 2019). Several genes connected to HLD have been discovered as a result of technological advancements, falling costs, and the integration of molecular genetic testing into clinical practice. The pathogenic variants in the *RARS1* gene (MIM#107820), like the majority of these genes, lead to a lack of myelin structural protein synthesis. (Kevelam et al., 2016; Pouwels et al., 2014; Wolf et al., 2021).

As earlier research has demonstrated, pathogenic variants of *RARS1* are strongly related to HLD and symptoms such as nystagmus, ataxia, and spasticity (Mendes et al., 2020). According to research on tRNA synthetase diseases, aminoacylation errors are linked to cell dysfunction. However, there is limited information regarding the pathogenic mechanism of *RARS1* variants (Mendes et al., 2020). Currently, due to the latest advances in molecular testing, the majority of patients have been genetically identified, with only around 10% remaining undiagnosed. Even in rare cases with only a few patients, exome sequencing (ES) has identified a defective gene (Rezaei et al., 2019; Wolf et al., 2014).

In this paper, we present three Bakhtiari patients in Iran with HLD9 who had homozygous *RARS1* pathogenic variants. In addition, we thoroughly reviewed the literature.

## 2 METHODS

### 2.1 Ethical compliance

**FIGURE 1:**
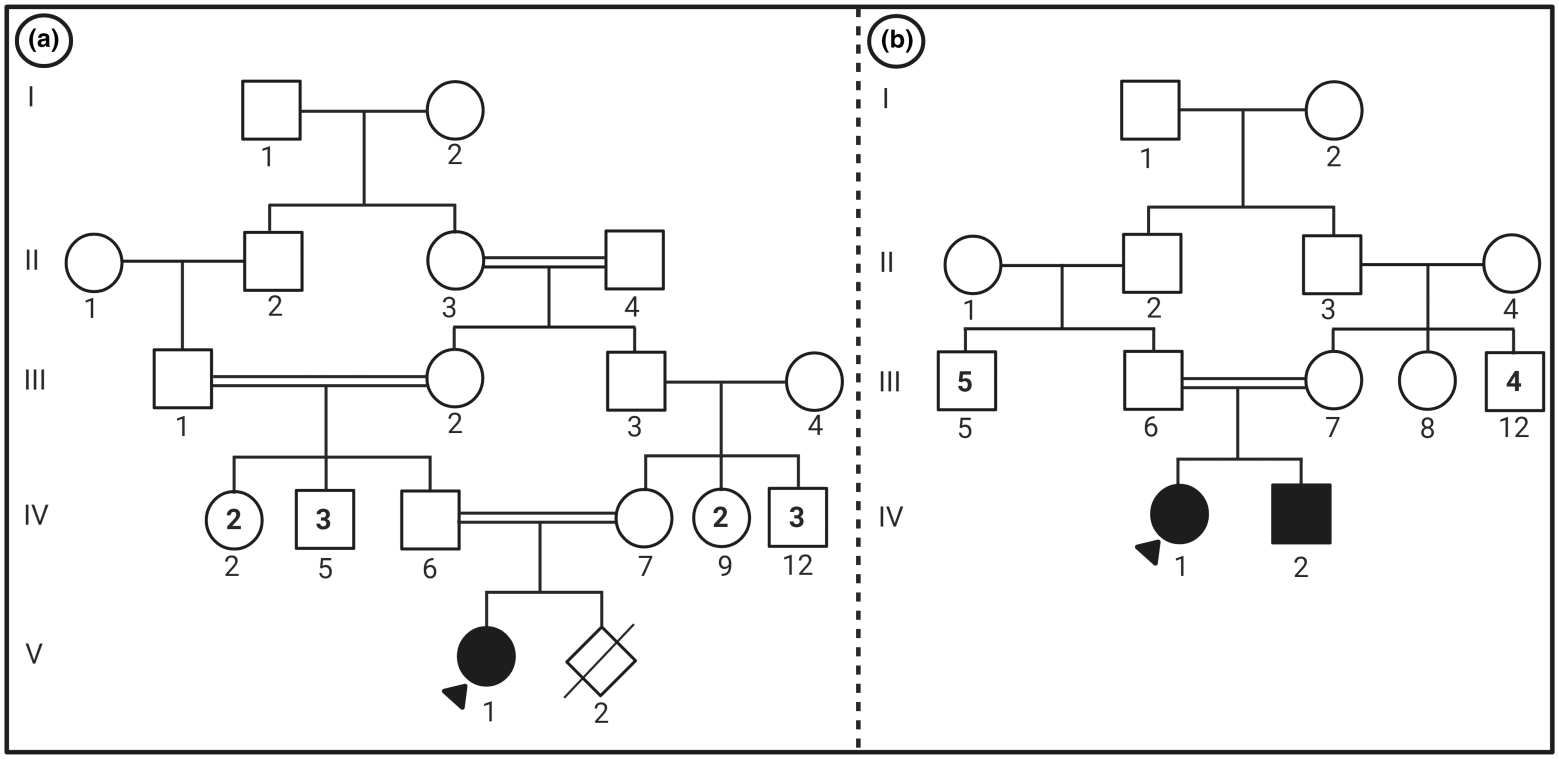
The patient's pedigrees. Family 1 (a), Family 2 (b).

The current study was approved by the Ethics Committee of the Isfahan University of Medical Sciences (IR.ARI.MUI.REC.1400.011). The research related to human use has complied with all the relevant national regulations, institutional policies, and in accordance with the tenets of the Helsinki Declaration and has been approved by the authors' institutional review board or equivalent committee. The pedigree of the HLD‐9 families with an autosomal recessive pattern of inheritance is shown in Figure 1.

### 2.2 DNA extraction and exome sequencing

After obtaining informed consent, DNA extraction from the peripheral blood of both patients and their families was performed using the salting‐out method. The genomic DNA samples were fragmented to construct the library for whole‐exome sequencing (WES) using the Agilent SureSelect v6 kit. The libraries obtained from the patients were sequenced to have >90× coverage on an Illumina Novaseq 6000 platform. In this study, sequencing was performed by the Illumina genome sequencing service in Macrogen (Seoul, Korea).

### 2.3 Bioinformatics analysis of exome data

The obtained sequences from both patients were aligned to the human reference genome (GRCh37/hg19) by the Burrows‐Wheeler‐Aligner (BWA). The variant calls are also generated using the Genomic Analyzer Toolkit (GATK) to identify variants relevant to the clinical indication. Using the Variant Effect Predictor (VEP) program, annotation of the variants was performed. Following this, variants were filtered using the available information from databases (such as HGMD, ClinVar, 1000 Genome, ExAC, LSDBs, dbSNP, and the locally available database, Iranome), published literature, clinical correlation, and its predicted function.

## 3 RESULTS

### 3.1 Case presentation

Case 1 was a 3‐year‐old female, the result of a consanguineous marriage with a history of Delayed psychomotor development, Dysarthria, Intellectual disability, symptomatic epilepsy, and microcephaly. The 1‐year‐old MRI of the patient revealed severe generalized atrophy, enlargement of subarachnoid space at the frontotemporal region, and mild communicating hydrocephalus.

Case 2 was a 6‐year‐old female from another consanguineous marriage. The reported symptoms of this patient were spasticity, developmental delay, microcephaly, and myoclonic seizures. The brain MRI for this patient revealed mild hypomyelination and enlargement of subarachnoid spaces in frontal regions. This patient has a 9‐year‐old brother (Case 3) with similar symptoms. These two families were unrelated and were referred to the Hope Generation Foundation Genetic Diagnosis Center simultaneously.

### 3.2 Mutational analysis

In both cases, WES showed a homozygous variant (NM_002887.4: c.2T>C) in *RARS1* (#MIM: 616140) that was consistent with the disease phenotype of patients. This start loss mutation causes the loss of the initiating codon and contemporary production of no protein. Sanger sequencing using specific primers for the variant‐containing region, the ES results were confirmed in all three patients. In silico study of the c.2T>C indicated the pathogenicity of this variant. The detected variant was not reported in ExAC and 1000 genomes and locally database, Iranome.

The detected variant was predicted to be damaging with different pathogenicity predictive tools (Table 1). It is predicted to result in a loss of the normal protein function. According to the ACMG guideline of variant classification (Richards et al., 2015), the identified variant was classified as ‘pathogenic’ because of having these criteria for the pathogenicity: PVS1 (null variant), PS1 (Same amino acid change as a known pathogenic variant), PM2 (Extremely low frequency in gnomAD population databases), PP1 (cosegregation), and PP4 (patient's phenotype or family history is highly specific for a disease with a single genetic etiology).

**TABLE 1:** Bioinformatics software prediction of the deleteriousness of the *RARS1* variant found in this case study.

Genetic variant	SIFT	MT^a^	DANN	BayesDel	gnomAD v4.0.0 frequency (Hom^b^)	Pathogenicity line of evidence based on ACMG
c.2T>C	Deleterious	Deleterious	Deleterious	Deleterious	0.000003	PVS1, PS1, PM2, PM3, PP1, PP4

^a^ Mutation taster.

^b^ Homozygous.

The combined annotation‐dependent depletion (CADD) score was 24.4, well above the mutation significance cutoff (MSC) of 24.2 (Itan et al., 2016; Kircher et al., 2014; Zhang et al., 2018).

### 3.3 Literature review and genotype/phenotype correlation

We searched PubMed, Scopus, Web of Science (science and social science citation index), and Google Scholar for articles published before November 2023. Article selection criteria included: (i) case reports of HLD9 due to *RARS1* variants; (ii) individuals have both defined variants and clinical manifestations; and (iii) language was limited to English.

Following the literature review, 25 different variants were identified in thirty HLD‐9 cases from eight studies. Variant c.5A>G (p.Asp2Gly) was present in 11 patients (Table 2). Surprisingly, 18 (55%) patients were compound heterozygous. Among the 66 variants in these 33 patients, missense was the major type with a frequency of 37, followed by start loss [14], splicing [7], and frameshift [7] (Table 2).

**TABLE 2:** Genotype of 33 patients with *RARS1* biallelic variants.

Case	Ethnicity	Age of onset	Subtype	Genotype	cDNA variant	Protein variant	Mutation type (coding impact)	Location	ACMG	CADD PHRED	Ref.
1	Dutch	12 months	Intermediate	Compound heterozygous	c.5A>G/c.45 + 1G>T	p.(Asp2Gly)/p.(?)	SNV (Missense)/SNV (Splicing)	Exon 1/intron 1	P/LP	23/33	(15)
2	Dutch	5 months	Intermediate	Compound heterozygous	c.5A>G/c.45 + 1G>T	p.(Asp2Gly)/p.(?)	SNV (Missense)/SNV (Splicing)	Exon 1/intron 1	P/LP	23/33	(15)
3	Dutch	10 months	Intermediate	Compound heterozygous	c.5A>G/c.96_97del	p.(Asp2Gly)/p.(Cys32Trpfs*39)	Missense/Del (Frameshift)	Exon 1/Exon 2	P/P	23/−	(15)
4	Dutch	2 months	Severe	Compound heterozygous	c.1A>G/c.1535G>A	p.(Met1?)/p.(Arg512Gln)	SNV (Start loss)/SNV (Missense)	Start codon defect/Exon 13	P/P	26/26	(15)
5	ND	3 months	Intermediate	Compound heterozygous	c.5A>G/c.1625 + 2T>G	p.(Asp2Gly)/p.(?)	SNV (Missense)/SNV (Splicing)	Exon 1/intron 13	P/LP	23/35	(22)
6	Maltese	12 months	Mild	Homozygous	c.5A>G	p.(Asp2Gly)	SNV (Missense)	Exon 1	P	23	(7)
7	Maltese	12 months	Mild	Homozygous	c.5A>G	p.(Asp2Gly)	SNV (Missense)	Exon 1	P	23	(7)
8	Turkish	3 months	Severe	Compound heterozygous	c.1367C>T/c.1846_1847del	p.(Ser456Leu)/p.(Tyr616Leufs*6)	SNV (Missense)/Del (Frameshift)	Exon 12/Exon 14	LP/LP	25/−	(7)
9	ND	8 months	Severe	Homozygous	c.1588A>G	p.T530A	SNV (Missense)	Exon 13	VUS	23	(23)
10	Iranian	1 month	Severe	Homozygous	c.2T>C	p.Met1Thr	SNV (Start loss)	Exon 1	P	24	(16)
11	Iranian	1 month	Severe	Homozygous	c.2T>C	p.Met1Thr	SNV (Start loss)	Exon 1	P	24	(16)
12	ND	3 months	Severe	Homozygous	c.1316C>A	p.(Ala439Asp)	SNV (Missense)	Exon 11	LP	26	(14)
13	ND	Congenital	Severe	Homozygous	c.1316C>A	p.(Ala439Asp)	SNV (Missense)	Exon 11	LP	26	(14)
14	ND	Congenital	Severe	Compound heterozygous	c.173T>C/c.1790T>C	p.(Leu58Pro)/p.(Leu597Pro)	SNV (Missense)	Exon 2/Exon 14	VUS/VUS	27/29	(14)
15	ND	10 months	Intermediate	Compound heterozygous	c.5A>G/c.1874‐9_1874‐5del	p.(Asp2Gly)/p.(?)	SNV (Missense)/SNV (Splicing)	Exon 1/intron 14	P/VUS	23/−	(14)
16	ND	10 months	Intermediate	Compound heterozygous	c.5A>G/c.1874‐9_1874‐5del	p.(Asp2Gly)/p.(?)	SNV (Missense)/SNV (Splicing)	Exon 1/intron 14	P/VUS	23/−	(14)
17	ND	36 months	Mild	Homozygous	c.5A>G	p.(Asp2Gly)	SNV (Missense)	Exon 1	P	23	(14)
18	ND	Congenital	Severe	Homozygous	c.67_70del	p.(Thr23Leufs*6)	Del (Frameshift)	Exon 2	LP	‐	(14)
19	ND	Congenital	Severe	Homozygous	c.67_70del	p.(Thr23Leufs*6)	Del (Frameshift)	Exon 2	LP	‐	(14)
20	ND	9 months	Intermediate	Compound heterozygous	c.668G>A/c.1568 T>A	p.(Arg223His)/p.(Met523Lys)	SNV (Missense)	Exon 6/Exon 13	VUS/VUS	29/26	(14)
21	ND	18 months	Mild	Compound heterozygous	c.5A>G/c.173T>C	p.(Asp2Gly)/p.(Leu58Pro)	SNV (Missense)	Exon 1/Exon 2	P/VUS	23/27	(14)
22	ND	1.5 month	Severe	Compound heterozygous	c.2T>A/c.448_456del	p.Met1?/p.(Cys150_Glu152del)	SNV (Start loss)/Del (Inframe)	Exon 1/Exon 5	P/LP	24/−	(14)
23	ND	Congenital	Severe	Compound heterozygous	c.2T>C/c.1535G>A	p.Met1Thr/p.(Arg512Gln)	SNV (Start loss)/SNV (Missense)	Exon 1/Exon 13	P/P	24/26	(14)
24	ND	2 months	Severe	Compound heterozygous	c.1452 + 1G>A/c.1534C>T	p.(?)/p.(Arg512Trp)	SNV (Splicing)/SNV (Missense)	Intron 13/Exon 13	P/LP	34/28	(14)
25	ND	Congenital	Severe	Compound heterozygous	c.1452 + 1G>A/c.1534C>T	p.(?)/p.(Arg512Trp)	SNV (Splicing)/SNV (Missense)	Intron 13/Exon 13	P/LP	34/28	(14)
26	ND	Congenital	Severe	Compound heterozygous	c.3G>T/c.96_97del	p.(Met1?)/p.(Cys32Trpfs*39)	SNV (Start loss)/Del (Frameshift)	Exon 1/Exon 2	P/P	25/−	(14)
27	ND	24 months	Mild	Compound heterozygous	c.475C>T/c.1367C>T	p.(Pro159Ser)/p.(Ser456Leu)	SNV (Missense)	Exon 5/Exon 12	VUS/LP	27/25	(14)
28	ND	3 months	Severe	Compound heterozygous	c.1535G>A/c.1382G>A	p.Arg512Gln/p.Arg461His	SNV (Missense)	Exon 13/Exon 12	P/VUS	26/26	(24)
29	ND	3 months	Severe	Homozygous	c.5A>T	p.(Asp2Gly)	SNV (Missense)	Exon 1	P	23	(24)
30	ND	ND	Mild	Homozygous	c.5A>G	p.(Asp2Gly)	SNV (Missense)	Exon 1	P	23	(21)
31	Iranian	Congenital	Severe	Homozygous	c.2T>C	p.Met1Thr	SNV (Start loss)	Exon 1	P	24	Current study Patient 1
32	Iranian	Congenital	Severe	Homozygous	c.2T>C	p.Met1Thr	SNV (Start loss)	Exon 1	P	24	Current study Patient 2
33	Iranian	Congenital	Severe	Homozygous	c.2T>C	p.Met1Thr	SNV (Start loss)	Exon 1	P	24	Current study Patient 3

The mean age of onset for HLD9 in the group of 33 patients with a known age of onset was 5.8 months (SD = 8.1), with a median age of onset of 3 months. The interquartile range of age of onset was 0–10 months. The age range of HLD9 in these patients was congenital to 36 months.

Among the 33 patients carrying the *RARS1* biallelic variants (including the three reported in this study), 17 (54%) were male patients, and 15 (46%) were female patients (one gender was not reported) (Table 3). Parts of Tables 2 and 3 had been carried over from the previous research (21). Among the 33 patients, 20 (60%) exhibited severe, 7 (22%) intermediate, and 6 (18%) mild phenotypes. Of the patients whose phenotype was reported, 22 (76%) had nystagmus, 31 (89%) had intellectual disability or developmental delay, 23 (89%) had language delay (17 (68%) with no speech), 25 (87%) had motor delay, 20 (69%) had microcephaly, 21 (70%) had atrophy, and 27 (94%) had hypomyelination.

**TABLE 3:** Phenotype of 33 patients with *RARS1* biallelic variants.

Case	Sex	Nystagmus	Epilepsy	Intellectual disability or developmental delay	Language delay	Speech absence	Highest motor milestone	Microcephaly	Main MRI findings		Ref.
									Hypomyelination	Cerebral atrophy	
1	F	+	_	+	_	_	Walks with support	_	+	_	(15)
2	F	+	_	+	+	_	Sits without support; crawls	_	+	_	(15)
3	M	+	_	+	+	_	Walks with support	_	+	_	(15)
4	F	+	_	+	+	+	Rolls over	+	+	+	(15)
5	F	+	+	+	ND	ND	ND	ND	+	+	(22)
6	M	+	_	+	+	_	Walks without support	ND	+	+	(7)
7	F	+	_	+	_	_	Walks without support (2.5 years)	ND	ND	ND	(7)
8	M	+	+	+	+	+	Sitting without support (2 years)	+	+	+	(7)
9	M	ND	ND	+	ND	ND	ND	+	ND	ND	(23)
10	F	+	_	+	+	ND	Head control (lost age 7 months)	+	+	_	(16)
11	M	+	+	+	ND	ND	Head control	+	+	+	(16)
12	M	+	+	+	+	+	Partial head control	+	+	+	(14)
13	M	_	+	+	+	+	Partial head control	+	ND	ND	(14)
14	M	+	+	+	+	+	No milestones achieved	+	+	+	(14)
15	M	+	_	+	+	+	Sits without support	_	+	+	(14)
16	M	+	_	+	+	+	Sits without support	_	+	+	(14)
17	ND	+	_	+	+	_	Walks without support	_	+	+	(14)
18	F	+	+	+	+	+	Partial head control	+	+	+	(14)
19	F	+	+	+	+	+	Partial head control	+	+	+	(14)
20	F	_	_	+	+	+	Walks without support	_	_	_	(14)
21	F	_	_	_	+	_	Walks without support	+	+	_	(14)
22	M	_	+	+	+	+	Partial head control	+	+	+	(14)
23	M	+	+	+	+	+	Rolls over, partial head control	+	+	+	(14)
24	M	_	+	+	+	+	No milestones achieved	+	+	+	(14)
25	M	+	+	+	+	+	No milestones achieved	+	+	+	(14)
26	F	_	+	+	+	+	No milestones achieved	+	+	+	(14)
27	F	_	_	_	+	+	Walks without support	_	_	_	(14)
28	F	+	+	+	ND	ND	Partial head control, Rolls over	+	+	_	(24)
29	M	+	_	+	+	+	Sits without support	_	+	_	(24)
30	M	+	ND	+	_	_	Walks without support	ND	ND	+	(21)
31	F	ND	+	+	ND	ND	ND	+	+	+	Current study Patient 1
32	F	ND	+	+	ND	ND	ND	+	+	+	Current study Patient 2
33	M	ND	+	+	ND	ND	ND	+	+	+	Current study Patient 3

## 4 DISCUSSION

We identified that *RARS1* homozygous variants can cause a variety of neurological symptoms, which is consistent with earlier findings (Di Bella et al., 2021; Ji et al., 2018; McSherry et al., 2018; Mendes et al., 2020; Nafisinia et al., 2017; Rezaei et al., 2019; Wan et al., 2023; Wolf et al., 2014). In the prior study, the clinical presentation was divided into three categories: severe, intermediate, and mild. A severe clinical presentation denoted that the patient's symptoms, which were typically refractory epilepsy, began to manifest during the patient's first 3 months of life (Mendes et al., 2020). In our study, all patients presented a severe phenotype with congenital onset. Furthermore, a severe developmental delay was observed.

RARS1 encodes cytoplasmic arginyl‐tRNA synthetase, an essential part of the aminoacyl‐tRNA synthetase in relation to enzymes needed for protein synthesis, which binds each amino acid to its specific tRNA (Eriani et al., 1990; Kim et al., 2014; Yang et al., 2014). RARS protein is a monomeric enzyme that belongs to the aminoacyl‐tRNA synthetase class I. Studies identified ∼72 kDa and ∼60 kDa cytoplasmic RARS in human cells. The 72 kDa is required for its interactions with AIMP1 in the multisynthetase complex. The 60 kDa N‐terminal truncated format is free in the cytoplasm and has no interactions with the multisynthetase complex. According to earlier research, even when a functional 60 kDa RARS is present, the 72 kDa format is crucial for protein synthesis and cell growth in Chinese hamster ovary cells. Both the 72 and 60 kDa isoforms arise from a single mRNA via alternative AUG start codons, have identical catalytic characteristics, and contribute roughly equally to the total rate of RARS in vitro movement (Zheng et al., 2006).

Variants in the genes encoding cytoplasmic and mitochondrial tRNA synthetases have been associated with a variety of disorders, ranging from peripheral neuropathy to leukodystrophy (Wolf et al., 2014). Biallelic *RARS1* variants have been linked to hypomyelination leukodystrophy in 30 cases (Di Bella et al., 2021; Ji et al., 2018; McSherry et al., 2018; Mendes et al., 2020; Nafisinia et al., 2017; Rezaei et al., 2019; Wan et al., 2023; Wolf et al., 2014). In this study, we used ES on 2 Iranian patients, both had Bakhtiari ethnicity and a history of consanguineous marriage. The Bakhtiari are an Iranian Lur tribe. Chaharmahal, Bakhtiari, eastern Khuzestan, Lorestan, Bushehr, and Isfahan provinces are where most Bakhtiari inhabit. Bakhtiari people maintain their bloodlines intact and mostly marry within their own tribe due to the hardships of their lifestyle.

In this research, we detected the c.2T>C pathogenic variant of *RARS1* that causes hypomyelination leukodystrophy in two unrelated families. According to a study, all patients with RARS‐related hypomyelination had a mean age of disease manifestation between 6 and 12 months of age (Pouwels et al., 2014). However, our analysis of the literature for all patients who have been diagnosed with HLD9 shows that this range is congenital to 10 months. In AR diseases, compound heterozygous genotypes can occasionally be found. However, in 55% of cases, the HLD9 compound heterozygous genotype has been observed. This issue is presumably brought on by the fact that most of the patients were found in cultures where consanguineous marriage was uncommon.

Pyramidal symptoms, more acute in the legs than arms, abnormal activities, developmental delay, motor regression, nystagmus, ataxia, and head titubation were present. Early‐onset nystagmus is usually a clinical outcome of leukodystrophies with hypomyelination. In the first study, four patients with hypomyelination were shown to have variants within *RARS1* with MRI alterations that were identical to those described in classical X‐linked recessive hypomyelination leukodystrophy‐1. Patients have been described as having mild mental retardation, ataxia, dysarthria, severe leg spasticity, mild intention tremor, and stagnated motor development (Wolf et al., 2014). In the current study, patients demonstrated similar motor and expressive delay features, head titubation, an action tremor, an ataxic gait, and cognitive impairment.

Among severe patients like our patients, in addition to the variants that caused protein truncation, such as those carrying splicing and frameshift defects, there were also two missense variants [c.1535G>A and c.1316C>A] and our variant (c.2T>C, p.Met1?) that were already present in several of the patients. The position of these missense variants in the catalytic region of Arginyl‐tRNA synthetase is possibly one of the reasons they cause severe phenotypes. This data implies a possible link between these variants and severe features. The variant c.5A>G p (Asp2Gly), which has been identified as the most prevalent RARS1, impacts the second amino acid residue aspartate. This residue is located within the 72 amino acid N‐terminal domain and is exclusive to the longer ArgRS isoform. The c.5A>G variant is most likely associated with a mild or intermediate condition, as no patients with this variant exhibit the severe early‐infantile phenotype, and all patients with homozygous status for this variant have a mild phenotype. (Di Bella et al., 2021; Ji et al., 2018; Mendes et al., 2020; Nafisinia et al., 2017; Wolf et al., 2014). As a result of our variant (c.2T>C variant), it is anticipated that no adequate protein will be made as a consequence of initiating methionine loss. Alternatively, an adenine, thymine, or guanine (ATG) codon may be utilized as a beginning codon in a downstream sequence, which can cause the presentation of a protein with an unknown format. If cellular compensatory mechanisms endeavor to neutralize the consequences of this variant, the 73rd codon of *RARS1* would be an ATG. The 3D structure investigation of the truncated RARS enzyme due to the c.2T>C missense variant offered a nonfunctional sequence of the first 72 amino acids in a truncated protein (Rezaei et al., 2019).

In conclusion, we report two patients with HLD9. The study also provides new insight into the disease spectrum linked to *RARS1* variants. The clinical characteristics of our patients, the position of the *RARS* variant, and our in silico analysis suggest that the RARS variant identified in this study can explain them.

## AUTHOR CONTRIBUTIONS

Sajjad Biglari was involved in the recruitment of patient and family members, mutation screening and segregation analysis in families by Sanger sequencing, and writing the first draft of the manuscript; Hassan Vahidnezhad, Mohammad Amin Tabatabaiefar, and Hamid Reza Khorram Khorshid were involved in writing the first draft of the manuscript and manuscript revision; Emran Esmaeilzadeh was involved in the conceptualization and supervision of the research, review and editing the first draft of the manuscript. All of the authors read and approved the final manuscript to be published and agreed to be responsible for the accuracy of the data and details.

## FUNDING INFORMATION

This study was supported by the Research Council of Hope Generation Foundation.

## CONFLICT OF INTEREST STATEMENT

The authors declare no conflict of interest.

## Data Availability

The data that support the findings of this study are available from the corresponding author upon reasonable request.
